# Post-Transbronchial Microwave Ablation Bronchopleural Fistula—A Case Series and Unique Insight

**DOI:** 10.1093/ejcts/ezaf456

**Published:** 2026-05-08

**Authors:** Aliss T C Chang, Cindy S Y Cho, Joyce W Y Chan, Wei Liu, Rainbow W H Lau, Calvin S H Ng

**Affiliations:** Division of Cardiothoracic Surgery, Department of Surgery, The Chinese University of Hong Kong, Prince of Wales Hospital, Sha Tin, N.T., Hong Kong, China; Division of Cardiothoracic Surgery, Department of Surgery, The Chinese University of Hong Kong, Prince of Wales Hospital, Sha Tin, N.T., Hong Kong, China; Division of Cardiothoracic Surgery, Department of Surgery, The Chinese University of Hong Kong, Prince of Wales Hospital, Sha Tin, N.T., Hong Kong, China; Division of Cardiothoracic Surgery, Department of Surgery, The Chinese University of Hong Kong, Prince of Wales Hospital, Sha Tin, N.T., Hong Kong, China; Division of Cardiothoracic Surgery, Department of Surgery, The Chinese University of Hong Kong, Prince of Wales Hospital, Sha Tin, N.T., Hong Kong, China; Division of Cardiothoracic Surgery, Department of Surgery, The Chinese University of Hong Kong, Prince of Wales Hospital, Sha Tin, N.T., Hong Kong, China

**Keywords:** transbronchial microwave ablation, bronchopleural fistula, navigation bronchoscopy, robotic-assisted bronchoscopy, minimally invasive therapy

## Abstract

**Objectives:**

This case series aims to evaluate the incidence, mechanisms, and management of bronchopleural fistula (BPF) following transbronchial microwave ablation (TMWA) for lung tumours and to explore innovative strategies for prevention and treatment.

**Methods:**

A retrospective review was conducted on 173 patients who underwent 209 sessions of TMWA from March 2019 to May 2025 at a single centre. Four cases of BPF confirmed by imaging and clinical presentation were analysed. Data collected included procedural details, mechanisms of BPF formation, management strategies, and patient outcomes. Techniques such as intraoperative fibrin glue injection and endobronchial valve placement were documented.

**Results:**

BPF occurred in 4 patients (1.9%) and was associated with mechanisms including extensive ablation zone with cavitation, tissue contraction, and inadvertent pleural puncture. Treatments varied from conservative drainage and antibiotics to targeted endobronchial interventions, with all BPF successfully resolved. The use of innovative techniques, such as intraoperative fibrin glue injection, demonstrated promising results with minimal invasiveness. Patients with BPF experienced longer hospital stays compared to those without complications.

**Conclusions:**

Although rare, BPF is a significant complication after TMWA, often requiring individualized management. Early recognition through vigilant monitoring and advanced imaging facilitates prompt intervention. Further prospective studies are needed to refine prevention and management strategies for this serious complication.

## Graphical abstract

**Figure:**
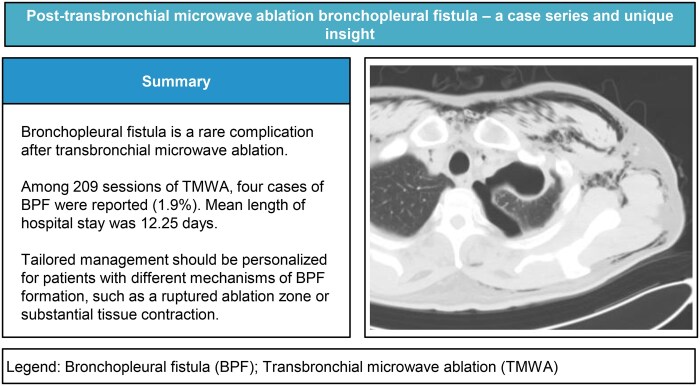

## Introduction

Early-stage and multifocal lung tumours are becoming a significant healthcare burden worldwide, especially with the widespread use of low-dose CT. While traditional major anatomical lung resection remains the gold standard of care, the demand for local treatment for these tumours is on the rise for many patients who are medically inoperable due to medical comorbidities or have inadequate pulmonary function.^1^ Local ablative therapy by percutaneous thermal ablation^2^ is one of the modalities used to achieve a satisfactory local disease control while minimizing loss of pulmonary function, maintaining quality of life, and avoiding major operative risks.

Despite the advantages of percutaneous thermal ablation, complications from local ablation can occur, mainly due to systemic reactions to post-ablation local inflammation and direct pleural puncture. For example, pleural-based complications such as pneumothorax and bronchopleural fistula (BPF) have been reported in the literature.^3^^,^^4^ Particularly, BPF remains a debilitating complication that has been associated with significant morbidity and poses substantial challenges in clinical management.^5^^,^^6^

Recently, transbronchial microwave ablation (TMWA) has emerged as a novel technique for the treatment of lung tumours, especially in multifocal tumours and metastases, due to its ability to treat concomitant lesions as well as very peripheral tumours in a single procedure and its adaptability to fit into a comprehensive multidisciplinary management pathway.^7^^,^^8^ This approach further minimizes the invasiveness of the procedure and offers comparable treatment efficacy to other local treatment modalities.

As TMWA avoids direct pleural puncture of the lungs, it carries a lower risk of pleural-based complications. However, the delivery of thermal energy can still lead to thermal injury in surrounding tissue outside of the tumour, which can result in complications such as BPF. This case series aims to explore the incidence and management of BPF following TMWA, shedding light on the potential mechanism behind BPF formation and highlighting the management strategies.

## Patients and methods

This is a single-centre retrospective review conducted from clinical data collected from March 2019 to May 2025, involving 173 patients who underwent TMWA. The indications for TMWA were either biopsy-confirmed malignant lung nodules or highly suspicious lung nodules. All cases have been submitted to the thoracic multidisciplinary meeting for comprehensive assessment to determine the presence of suspicious features and relevant oncological background, and to assess suitability and feasibility for TMWA. Whole-body positron emission tomography (PET) is mandatory, and nodal staging with endobronchial ultrasound is performed as needed. All post-TMWA BPF cases within the study time frame have been included in this case series, which comprises 4 cases confirmed by radiological imaging. A retrospective review of the medical records was carried out in these 4 cases. This study was approved by the Joint Chinese University of Hong Kong—New Territories East Cluster Clinical Research Ethics Committee.

All patients underwent a preoperative CT of the thorax for route planning. The TMWA was performed in the hybrid operating room under navigation bronchoscopy (either electromagnetic navigation bronchoscopy or robotic-assisted bronchoscopy) as per our routine protocol. Once the navigation was deemed successful using intraoperative imaging, the ablation catheter was introduced for the ablation. Cone-beam CT (CBCT) and augmented fluoroscopy were used intraoperatively as imaging adjuncts to confirm optimal tool position, to assess the adequacy of the ablation zone coverage and margins, and to evaluate for any intraoperative complications. In cases of inadequate coverage or insufficient margins on CBCT, re-ablation can be performed by the same spot re-ablation, pull-back re-ablation, or re-navigation for bracket re-ablation, as per our routine practice as described in the previous literature.^9^

The first postoperative chest X-ray (CXR) was taken immediately after the procedure. Clinical assessment and CXR were performed daily throughout the in-hospital stay. Upon discharge, the first postoperative outpatient visit was scheduled 2 weeks later, with CXR and clinical assessment performed during the visit. Afterwards, all patients were followed up with either CT or PET at 1, 3, 6, and subsequently every 6 months.

Descriptive statistics were used. Categorical variables were presented as crude count and frequency (%).

## Results

### Patient and procedure characteristics

Between March 2019 and May 2025, 173 patients received 209 sessions of TMWA, and a total of 281 lung nodules were treated at our institution. **Table 1** shows the patient and nodule characteristics from all patients (*N* = 173). Among the 209 TMWA sessions, 4 cases developed post-TMWA BPF (1.9%). Among the BPF cases (*N* = 4), the ages ranged from 57 to 81 (median age = 63). The indications for TMWA were suspicious multifocal lung tumours, medically inoperable lung adenocarcinoma, and oligometastases. In the 4 cases of post-TMWA BPF, a total of 6 lung nodules were treated with ablation, and technical success was achieved in all cases (100%), with a median minimum margin of 7 mm (IQR = 0). One case had concomitant video-assisted thoracoscopic surgery (VATS) for additional treatment of an ipsilateral peripheral lung cancer, while 2 cases had concomitant TMWA to 2 ipsilateral lung nodules. The median nodule size ablated was 10.5 mm (IQR = 3.85). The median minimal distance from the nearest pleura was 12.7 mm (IQR = 13.3). Five nodules were treated with single ablation alone, while one lung nodule required double ablation due to suboptimal ablation zone coverage after the initial ablation.

**Table 1.** Patient and Nodule Characteristics Among All 173 Patients

Patient characteristics (*N* = 173)	Median (IQR)/count (%)
Age	66 (14)
Male	73 (42.2%)
Female	100 (57.8%)
No COPD	160 (92.5%)
GOLD I COPD	6 (3.5%)
GOLD II COPD	6 (3.5%)
GOLD III COPD	1 (0.5%)

Nodule characteristics (*N* = 281)	Median (IQR)/count (%)
Size	10 mm (7 mm)
Left upper lobe	71 (25.3%)
Left lower lobe	50 (17.8%)
Right upper lobe	80 (28.5%)
Right middle lobe	28 (9.9%)
Right lower lobe	52 (18.5%)
No biopsy	200 (71.2%)
Non-small cell lung carcinoma	38 (13.5%)
Metastatic tumours	13 (4.6%)
Atypical adenomatous hyperplasia or adenocarcinoma in situ	30 (10.7%)

Abbreviation: COPD, chronic obstructive pulmonary disease.

### Mechanism and management

The observed mechanisms of BPF formation included significant post-ablation changes with expansion of cavitation, significant post-ablation changes with expansion and rupture of cavitation, and substantial tissue contraction with inadvertent pleural puncture by tools. All 4 cases were presented with increased pneumothorax or surgical emphysema, and all patients received postoperative CT for the diagnosis of BPF. The mean length of stay (LOS) was 12.25 days (range 2–20 days).

The following is the observed mechanism of BPF formation and the management of BPF for each patient. Patient and procedural details of the BPF cohort are summarized in **Table 2** (*N* = 4).

**Table 2.** Patient and Procedural Details of the BPF Cohort (*N* = 4)

Patient	Indications for TMWA	Mechanism of BPF formation	Clinical presentation	Management	Outcomes and follow-up
A	Oligometastases	Rapidly expanded cavitatory ablation zone	Increased pneumothorax on POD 1	Pleural drainage with antibiotic	Total LOS = 16 days; BPF healed with contracted ablation zone
B	Highly suspicious multifocal lung tumours	Rapidly expanded and ruptured ablation zone	Increase pneumothorax and surgical emphysema on POD 2	Pleural drainage and EBV placement	Air leak stopped immediately after EBV; total LOS = 11 days; EBV retrieval 8 weeks after placement; BPF healed with a largely scarred-down ablation zone
C	Medically inoperable adenocarcinoma	Significant tissue contraction and positional shift of the ablation catheter	Developed pneumothorax immediately after the operation and readmission for recurrent pneumothorax on POD 8	Pleural drainage with EBV placement	Air leak stopped immediately after EBV; total LOS = 20 days; EBV retrieval 6 weeks after the placement; BPF healed with contracting ablation zone
D	Highly suspicious of multifocal lung tumours (history of contralateral lobectomy for lung adenocarcinoma)	Inadvertent breach in the pleural from instrument manipulation and significant tissue contraction, leading to tool protrusion into the pleural cavity	Intraoperative detection of pneumothorax on CBCT and fluoroscopy	Intraoperative transbronchial fibrin glue injection	Postoperative CXR showed resolving pneumothorax; total LOS = 2 days; BPF healed

Abbreviations: BPF, bronchopleural fistula; CBCT, cone-beam CT; CXR, chest X-ray; EBV, endobronchial valve; LOS, length of stay; POD, postoperative day; TMWA, transbronchial microwave ablation.

**Figure 1.**
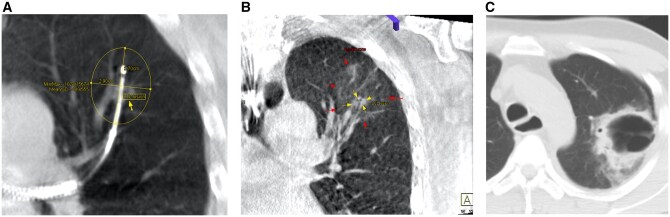
Patient A intraoperative CBCT and postoperative CT. (A) Intraoperative CBCT before TMWA showed a semi-solid cavitatory oligometastases and an optimal ablation catheter placement, with measurement of predicted ablation zone. (B) Intraoperative CBCT performed after ablation showed an adequate ablation zone covering the lesion with ample margins and no pleural complications. (C) Postoperative CT of the thorax was performed in view of the new onset of pneumothorax. CT showing an extensive ablation change with a large cavitation and a small amount of pleural effusion. A small fistula was noted at the periphery. Abbreviations: CBCT, cone-beam CT; TMWA, transbronchial microwave ablation.

Patient A received TMWA for a cavitatory lung metastasis in the left upper lobe (**Figure 1A**), and TMWA was performed uneventfully (**Figure 1B**). Pneumothorax was not evident on initial CBCT or CXR, but was seen on postoperative day (POD) 1 CXR, while the patient remained asymptomatic. CT was performed on POD 2, showing a rapidly expanded cavitatory ablation zone with fistulation at the periphery and a small amount of fluid within the cavitatory ablation zone and the pleural cavity (**Figure 1C**). He received pleural drainage for both the pneumothorax and the small amount of pleural effusion. Airleak from the chest drain was present and stopped after 5 days. Later, the bacterial culture of the pleural fluid returned positive for a Bacillus species microorganism, which was treated with a course of intravenous antibiotics. The pleural fluid became sterile 4 days after drainage, and the chest drain was removed on POD 16. The total LOS for patient A was 16 days.

**Figure 2.**
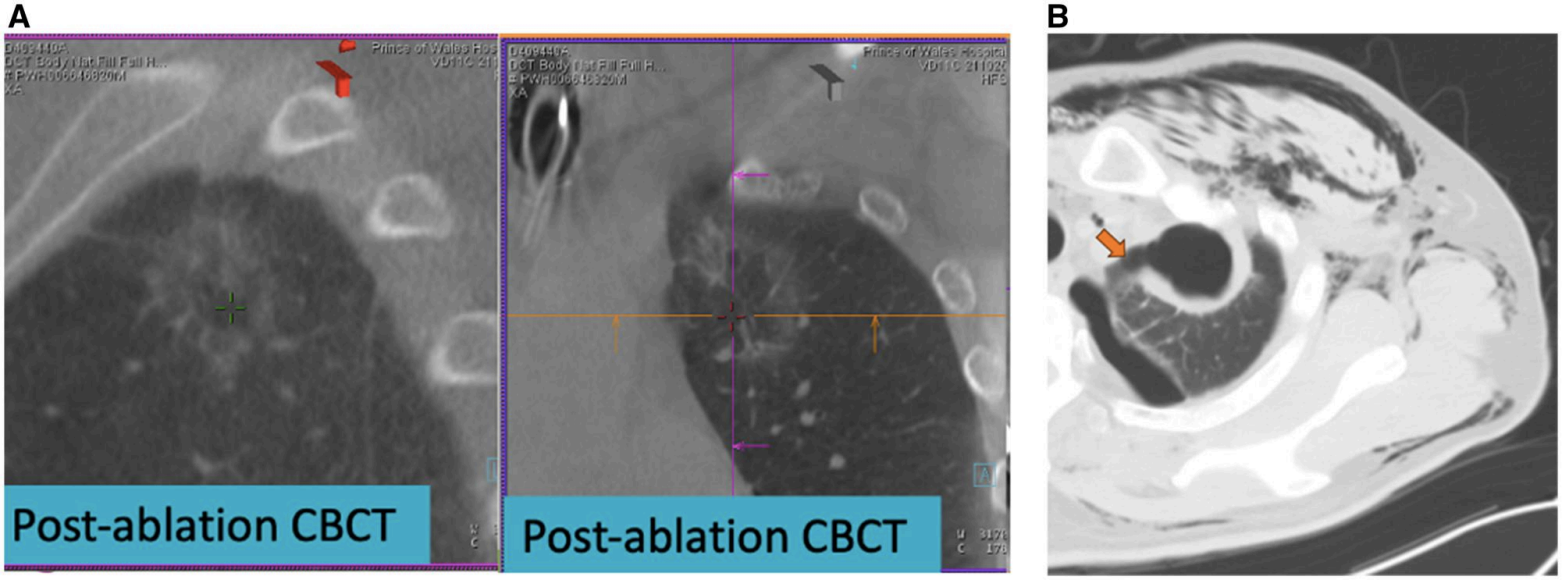
Patient B Intraoperative CBCT and postoperative CT. (A) Intraoperative CBCT showing successful ablation of both lung lesions with adequate margins. No immediate pneumothorax was noted on intraoperative imaging. (B) Postoperative CT of the thorax was performed in view of increased air leakage from the chest drain and increased surgical emphysema. The CT showed an extensive ablation zone with large cavitation and a ruptured ablation zone. Abbreviation: CBCT, cone-beam CT.

Patient B received concomitant TMWA for 2 suspicious multifocal lung tumours in the left upper lobe (**Figure 2A**) and same-session ipsilateral 2-port VATS segmentectomy for a proven lung cancer in the left lower lobe. A chest tube was placed at the end of the VATS. He was found to have increased pneumothorax and emphysema on POD 2, and a diagnostic CT was performed, which showed a rapidly expanded and ruptured cavitatory ablation zone (**Figure 2B**). Flexible bronchoscopy was done on POD 9 to assess the feasibility of endobronchial valve (EBV) placement given significant air leakage. Eventually, 2 EBVs were placed to block off the corresponding left upper lobe segmental bronchi, and the air leakage ceased immediately after EBV placement. The chest tube was removed on POD 10, and the total LOS was 11 days.

**Figure 3.**
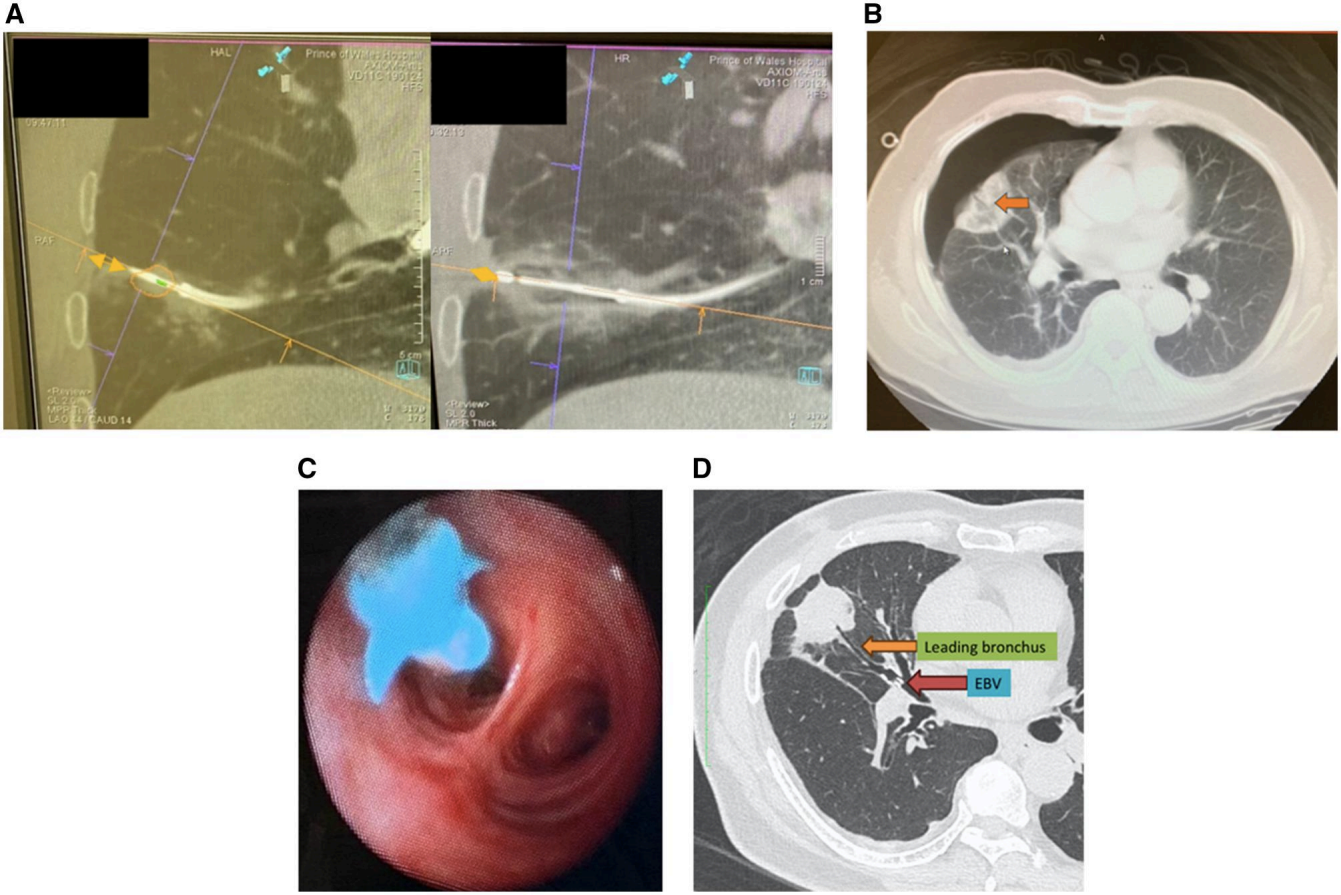
(A) Intraoperative CBCT showing significant tissue contraction and a shift in the position of the catheter. The catheter shifted closer to the visceral pleura after TMWA. (B) CT was performed as the patient developed a pneumothorax immediately after the procedure. The CT showed a single BPF following the ablation tract from a single leading airway (orange arrow). (C) Bronchoscopic EBV placement into the leading airway was performed. Air leak ceased immediately after EBV placement. (D) An interval CT was performed at 1 month, showing a contracted ablation zone and a healing BPF coming from the leading airway (orange arrow). EBV was in situ (red arrow). Abbreviations: BPF, bronchopleural fistula; CBCT, cone-beam CT; EBV, endobronchial valve; TMWA, transbronchial microwave ablation.

Patient C received TMWA for a biopsy-proven lung adenocarcinoma located near the pleura of the lateral segment of the right middle lobe (the nearest distance from the nearby pleura was 5.4 mm). During the procedure, post-ablation CBCT showed generous tissue contraction of the ablation zone, resulting in a slight shift in the position of the ablation catheter, and prompting suspicion of inadvertent pleura breached by the catheter (**Figure 3A**). However, no evident pneumothorax or protrusion of the catheter was detected on the intraoperative CBCT. Yet, this patient presented with a large pneumothorax with collapsed lung on the immediate postoperative CXR in the recovery room. Hence, a chest drain was inserted. Air leakage was stopped on POD 1, and the drain was removed on POD 2. The patient was discharged home after the drain was removed. However, he was noted to have recurrent pneumothorax on POD 8 and needed readmission for chest tube drainage, and a prolonged air leak was noted after a week of drainage. Eventually, the diagnosis of BPF was made on CT, showing a BPF from the leading airway where the ablation catheter was placed (**Figure 3B**). An elective flexible bronchoscopy was performed with an EBV placed to block off the leading airway (from the lateral segmental bronchus) 15 days after the readmission (**Figure 3C**). Air leakage stopped immediately after EBV placement, and the chest tube was weaned 2 days later (**Figure 3D**). Total LOS for patient C was 20 days.

**Figure 4.**
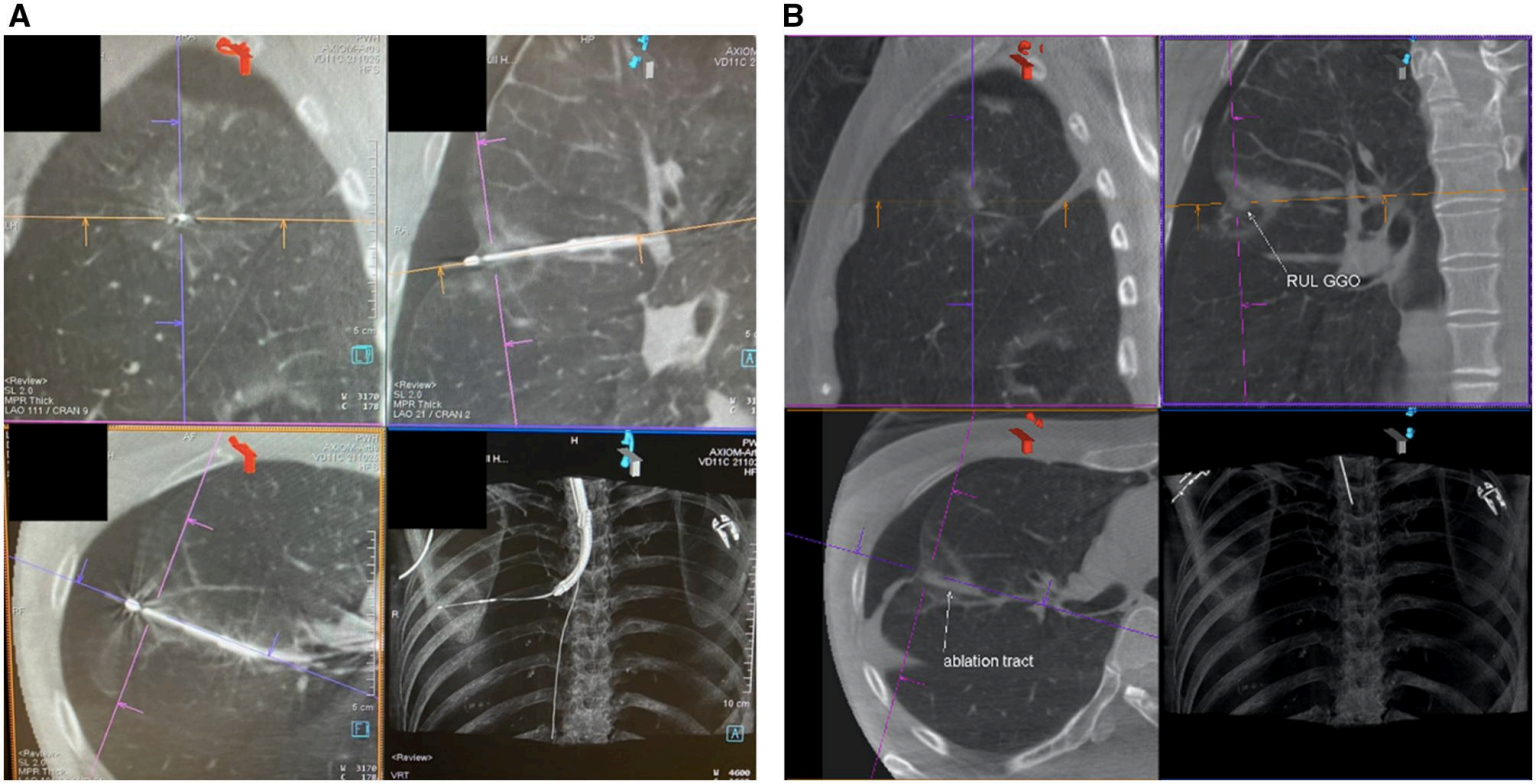
Patient D intraoperative CBCT. (A) Intraoperative fluoroscopy detected the shift in position of the ablation catheter. CBCT showed the catheter had shifted due to significant tissue contraction, resulting in protrusion into the pleural cavity. (B) Fibrin glue was injected along the ablation tract through the EWC, successfully sealing off the BPF. Abbreviations: BPF, bronchopleural fistula; CBCT, cone-beam CT; EWC, extended working channel.

Patient D received TMWA for 2 suspicious ipsilateral multifocal lung tumours, with a nearest distance to the nearby pleura of 6.9 mm. During the TMWA, a breach of the nearby pleura was created during instrument manipulation. The patient remained stable during the procedure; therefore, the decision was made to exchange to an ablation catheter to deliver the ablation. A substantial tissue contraction of the ablation zone was noted, which led to the protrusion of the tip of the ablation catheter into the nearby pleural cavity through the breach in pleura created prior (**Figure 4A**). A pneumothorax was also detected on intraoperative CBCT. As the ablation catheter remained in place during detection of a BPF and protruded into the pleural space, it was exchanged for an extended working channel (EWC), which served as a hollow catheter, allowing fibrin glue injection. Using the EWC, fibrin glue was injected along the BPF from the most peripheral to the most central segment of the airway by pulling the EWC back gradually during the injection (**Figure 4B**). Afterward, the EWC and bronchoscope were removed. Intraoperative imaging confirmed adequate sealing of the BPF with a static pneumothorax. No chest drainage was required for patient D. Postoperative CXR on POD 1 and 2 showed a reduced pneumothorax, and the total LOS for patient D was 2 days.

**Figure 5.**
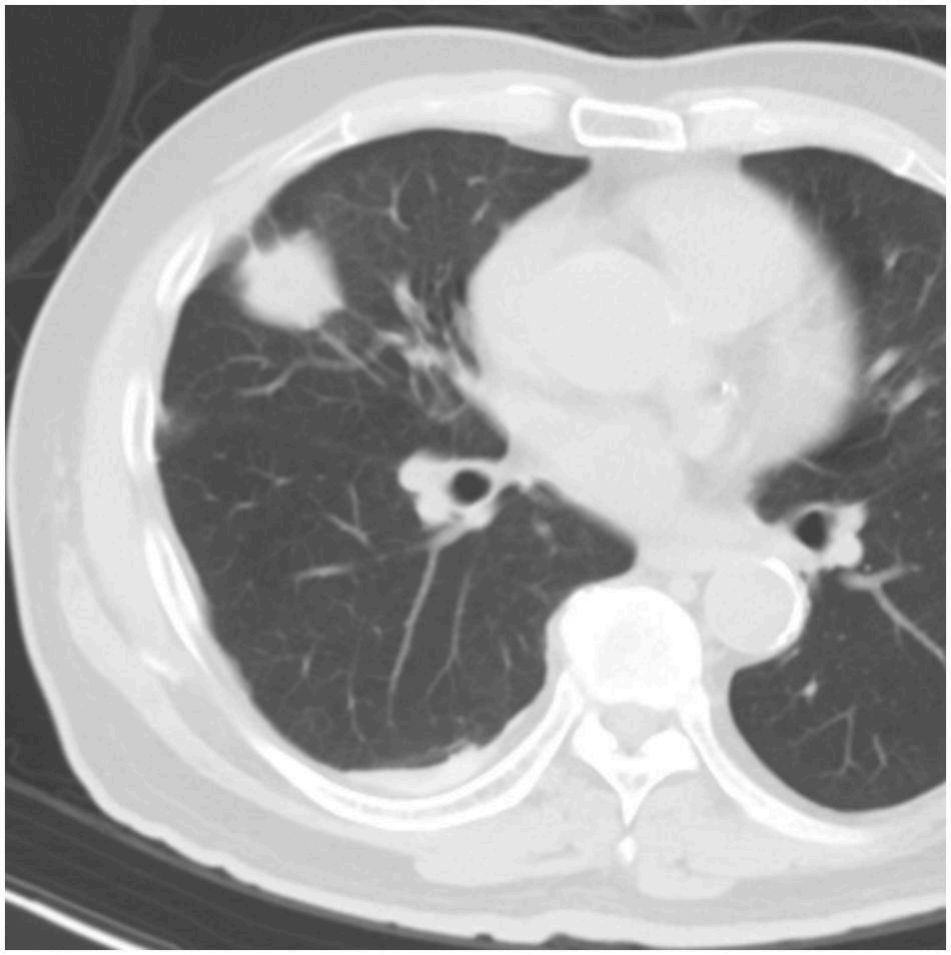
Follow-Up CT Scans Were Taken at 3 Months, Showing a Completely Healed BPF With Interval Contraction of the Ablation Zones. Abbreviation: BPF, bronchopleural fistula.

Follow-up CTs were performed in all 4 cases at 1- and 3-month intervals, which all showed healed BPF with well-formed and contracted ablation zones (**Figure 5**). For the 2 patients who received EBV placement, the EBVs were removed electively after 6 and 8 weeks, respectively.

## Discussion

Currently, there are limited clinical data on post-TMWA BPF in the existing literature. The findings from this case series underscore the complexity of BPF as a complication following TMWA. Previous literature on percutaneous lung ablation reported a 0.4%-2% incidence of BPF after percutaneous ablation.^4^^,^^6^ Even though BPF is rare, the morbidity and mortality related to BPF were high, with a reported mortality rate of 15%-70% as further infectious complications often occur.^10^^,^^11^ BPF often leads to prolonged hospital stays and requires intensive management, impacting patient recovery and healthcare resources. In this series, the incidence of BPF after TMWA remains rare, observed in only 1.9% of cases. No mortality related to the procedure or the presence of BPF was reported. However, the LOS of patients with BPF was significantly longer than that of those without BPF in a crude comparison (1.55 versus 12.25 days). This highlights the need for heightened awareness and strategic approaches to mitigate these risks in the context of TMWA.

A critical limitation of this case series is the inability to analyse potential risk factors specific to post-TMWA BPF formation, due to the small sample size. Nonetheless, previous studies have suggested several possible contributors to BPF formation following percutaneous lung ablation, which may also apply to TMWA. These include multiple pleural punctures, extensive ablation zones, and pre-existing pulmonary conditions such as chronic obstructive pulmonary disease (COPD) or emphysema.^3^ In this series, 2 potential primary mechanisms of BPF formation were observed, which might increase the risk of post-TMWA BPF. These include extensive ablative changes with rapidly expanded cavitation and significant tissue contraction.

Compared to percutaneous ablation, the heat transfer to the pleura is minimized in TMWA, as there is no direct pleural puncture. Thus, multiple ablations in TMWA alone would not necessarily increase the risk of BPF formation.^12^ This theory is demonstrated in a study cohort published by Chan et al, which includes patients who underwent concomitant TMWA for multiple lung nodules. They showed that concomitant TMWA up to 4 nodules did not increase the risk of complications compared with single-nodule ablations, and no BPF was reported despite multiple ablations.^12^ Moreover, an extensive ablation zone or substantial tissue contraction after TMWA might be a risk factor for BPF formation due to the unavoidable post-ablation lung tissue necrosis, which promotes BPF formation after the excretion of necrotic tissue. Significant tissue contraction can also lead to a positional shift of the transbronchial instruments, causing inadvertent puncture of the nearby pleura and creating BPF. A study cohort comparing TMWA in subpleural and deep lung nodules revealed that TMWA in subpleural lesions is safe and does not increase the overall risk of pneumothorax or BPF. However, pneumothorax was reported when incidental pleural puncture was observed, irrespective of the location of the nodules.^8^ Consequently, we hypothesize that BPF formation is related to the unexpected pleural puncture from tools, which is influenced by tissue contraction. Lastly, no direct correlation between COPD or emphysema and post-TMWA BPF can be concluded in this study. However, studies conducted for percutaneous microwave ablation, COPD, and emphysema are significant contributors to post-ablation BPF formation.^3^^,^^13^ One of the cases in this series involved TMWA of a cavitatory lesion, which may behave similarly to emphysema, as it also exhibits reduced ventilation and perfusion, and could affect the ablation effect. Multicentre prospective data will help further delineate the possible risk factors for post-TMWA BPF formation.

To manage BPF, a tailored management strategy should be adopted that accounts for the specific underlying mechanisms. Management should be individualized according to these mechanisms and adhere to the following principles. Firstly, it is essential to ensure adequate drainage of any pneumothorax and/or pleural effusion to prevent further respiratory complications. Secondly, effective air leak management tailored to the underlying mechanism is crucial to avoid prolonged drain placement and extended hospital stays. Lastly, preventing secondary complications, such as infection, and close monitoring for evolving complications are necessary. Tailored management may range from conservative measures, such as antibiotics and drainage alone in patients with small fistulas from weakened, cavitary ablation zones accompanied by pleural effusion, to more invasive interventions, such as EBV placement in patients with sizeable BPF from ruptured ablation zones.^14^ The use of EBV in the management of BPF or persistent air leak has been reported and shown to shorten LOS significantly.^15^ In this series, postoperative CT is used to visualize the fistula, and bronchoscopy is performed to confirm the culprit airway(s) with the balloon occlusion test. With both radiological and bronchoscopic confirmation, effective EBV placement can be ensured, and the degree of air leak can be monitored thereafter from the pleural drainage.

In this case series, one patient underwent intraoperative transbronchial fibrin glue injection for the management of BPF. This innovative approach is employed when the BPF is clearly identified via intraoperative imaging. As the associated BPF is readily accessible with transbronchial tools intraoperatively, an EWC or catheter can be easily exchanged and placed into the fistula tract for precise delivery of the therapeutic agent, achieving adequate fistula sealing and avoiding additional invasive procedures or future device retrieval. Fibrin glue, a surgical adhesive usually used for the management of mild to moderate bleeding, adheres rapidly to surrounding soft tissue and possesses sufficient viscosity to occlude the fistula without significant risk of proximal migration. Comparable techniques have been reported in case reports of BPF following percutaneous lung ablation, where surgical sealant or glue is percutaneously administered.^16^^,^^17^ These techniques have the potential to minimize the duration of air leak, shorten hospital stays, and carry a relatively low risk of associated complications. Intraoperative transbronchial fibrin glue injection can potentially obviate the need for chest drain insertion.

## Conclusion

TMWA represents a promising, minimally invasive approach for treating lung tumours, offering significant therapeutic benefits. Despite its advantages, TMWA carries the risk of serious complications such as BPF, which, although rare, can lead to prolonged hospital stays and increased patient morbidity. This article describes mechanisms of BPF formation and management options that can be used to manage BPF and its related complications. Innovative techniques, such as intraoperative transbronchial fibrin glue injection, hold promise for precise management of BPF. Multicentre data are essential for identifying risk factors and developing optimal management strategies for post-TMWA BPF, ultimately enhancing patient safety and treatment efficacy.

## Data Availability

The data underlying this article cannot be shared publicly due to the privacy of individuals who participated in the study. The data will be shared on a reasonable request to the corresponding author.
